# Insights Into Contemporary Practices in the Prehabilitation of Sarcopenic Patients Prior to Surgery: An Up-to-Date Review

**DOI:** 10.7759/cureus.88218

**Published:** 2025-07-18

**Authors:** Chenyi Mao, Madeline Rogers-Seeley, Samuel B Tay, Khang Duy Ricky Le

**Affiliations:** 1 General Surgery, University of Melbourne, Melbourne, AUS; 2 General Surgery, Royal Melbourne Hospital, Parkville, AUS; 3 Critical Care Medicine, Eastern Health Box Hill, Box Hill, AUS; 4 Surgery, Northeast Health Wangaratta, Wangaratta, AUS

**Keywords:** multicomponent exercise for sarcopenia, peri-operative care, prehabilitaiton, resistance exercise, severe sarcopenia

## Abstract

Sarcopenia is increasingly recognised as a key predictor of adverse peri-operative outcomes, including longer hospital stays, increased post-operative complications, delayed recovery, and greater mortality. Multimodal prehabilitation targeted at sarcopenia has therefore gained recognition as a method to improve outcomes; however, standardised, evidence-based prehabilitation regimens to optimise sarcopenic patients prior to surgery remain poorly defined and highly heterogeneous.

This review aims to synthesise current insights into contemporary multimodal prehabilitation approaches for patients with sarcopenia undergoing surgery, with a focus on improving clinical outcomes and reducing peri-operative risk.

The review explores five core domains of prehabilitation: resistance exercise, nutritional optimisation, psychological support, inflammation management, and hormonal regulation. Resistance training, particularly at moderate to vigorous intensity, has robust evidence for improving muscle strength and function. Nutritional strategies, especially protein and leucine supplementation, play a key role but are limited by variable dosing and adherence. Psychological support, increasingly integrated into prehabilitation, improves patient readiness and quality of life. Managing chronic inflammation and hormonal imbalances is emerging as a complementary strategy to enhance muscle health. The pathophysiology of sarcopenia also varies, with oncologic sarcopenia driven by systemic inflammation and cachexia, necessitating tailored interventions.

Given the ageing global population and rising surgical demand, effective prehabilitation for sarcopenic patients is critical. A multidisciplinary, individualised approach can help reduce variability in care, improve resilience to surgical stress, and ultimately enhance patient outcomes. Standardising these interventions within peri-operative pathways is a key step forward in modern surgical care.

## Introduction and background

Sarcopenia in the peri-operative period is becoming increasingly recognised as a significant predictor of poor surgical outcomes, including increased length of hospital stay, post-operative complication rates, morbidity, and mortality [1]. The European Working Group on Sarcopenia in Older People (EWGSOP2, 2019) defines sarcopenia as a “progressive and generalised skeletal muscle disorder associated with an increased likelihood of adverse outcomes including falls, fractures, physical disability, and mortality” [2]. Diagnosis should consider reduced muscle strength (as a primary indicator), low muscle quantity or quality, and impaired physical performance [2].

Despite the growing awareness of its clinical implications, there is a gap in the literature and practice regarding the structured, standardised implementation of prehabilitation to mitigate sarcopenia before surgery. This lack of consistency contributes to significant heterogeneity in peri-operative care and, similarly, varied results for patients. Moreover, the pathophysiology of sarcopenia differs between primary (age-related) and secondary forms. For instance, cancer-related sarcopenia is driven predominantly by systemic inflammation and metabolic dysfunction, and thus may require unique, targeted prehabilitation approaches [3].

As the global population continues to age [4], optimising peri-operative outcomes through evidence-based prehabilitation strategies for sarcopenic patients becomes increasingly critical. This review synthesises current evidence across key domains, exercise, nutrition, psychological support, inflammation management, and hormonal regulation, to inform contemporary best practices in prehabilitation for this high-risk group.

## Review

An overview of modern prehabilitation program structure

**Figure 1:**
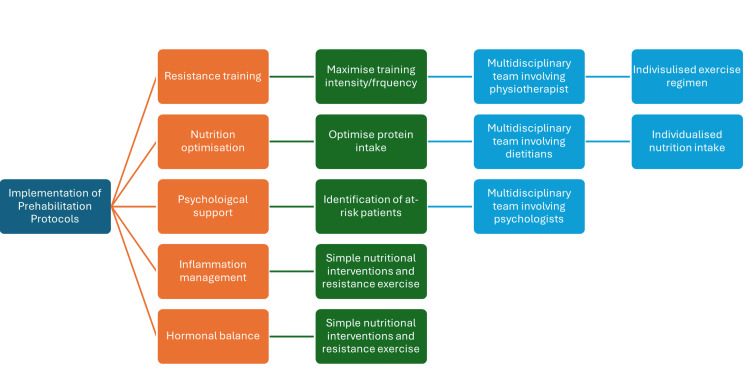
A multimodal prehabilitation program in patient with sarcopenia. This flowchart illustrates the structured implementation of prehabilitation protocols, highlighting five essential intervention domains: resistance training, nutrition optimisation, psychological support, inflammation management, and hormonal balance. Each domain is linked to practical strategies (e.g., optimising protein intake, maximising training intensity) and supported by a multidisciplinary team, including physiotherapists, dietitians, and psychologists. The ultimate goal is to deliver individualised exercise and nutritional regimens tailored to patient needs. This integrated approach addresses the multifactorial nature of sarcopenia and aims to improve surgical resilience and recovery outcomes.

With emerging evidence identifying sarcopenia as an important predictor of post-operative complications, multidisciplinary, multimodal prehabilitation regimens have been implemented to improve outcomes for surgical patients. Across the literature, optimal prehabilitation regimens remain poorly defined, exhibiting substantial heterogeneity in design and, consequently, in outcomes. Confounding variables such as comorbidities, age, functional limitations, and varying surgical risks further complicate characterisation [5]. Despite this, a few key areas are central to contemporary prehabilitation. For example, resistance training (RT), alongside balance and functional mobility training, has shown benefits in preventing the progression of sarcopenia, as well as reducing fall risk, particularly in frail or sarcopenic patients. Nutritional optimisation, focused on adequate protein and energy intake, is another cornerstone of management and is typically delivered in conjunction with exercise components to support muscle anabolism. Delivery models, however, vary depending on factors such as resource availability, cost, and feasibility. These models range from supervised in-hospital sessions to home-based and telehealth platforms, tailored to patient needs and healthcare infrastructure. Furthermore, current research on augmented prehabilitation highlights the importance of mental health interventions, hormonal balance management, and chronic inflammation control in improving patient outcomes [6]. We outline a multimodal approach to a prehabilitation program that can be utilised in patients with sarcopenia (Figure 1).

Insights into contemporary prehabilitation for sarcopenic patients prior to surgery

Exercise Training

Sarcopenic patients are suggested to have a 60% increased risk of falls, an 84% increased risk of fractures, and often exhibit reduced cardiorespiratory fitness [6, 7]. Accordingly, interventions for this group commonly adopt a multimodal exercise approach encompassing RT, balance training, and aerobic exercise. Among these, RT is considered the cornerstone of sarcopenia treatment [5]. The recommendation to apply RT is supported by robust evidence demonstrating improvements in muscle mass and strength, and it plays a key role in maintaining physical fitness and sports performance in middle-aged and older adults [8-11]. Most contemporary exercise regimens in the literature span three to twelve weeks and involve two to three sessions per week at moderate to high intensity, often 60% to 80% of one repetition maximum, in small groups (<12 participants) [12-14]. Exercises target major muscle groups using RT machines, free weights, or resistance bands, and may include chest press, seated row, leg extension, and curl and press exercises. However, no universal consensus exists on the optimal structure, duration, or intensity of pre-operative RT programs, contributing to variability in clinical practice.

The intensity of RT appears to be a key determinant of its effectiveness. In a meta-analysis, Chen YC et al. revealed that moderate-to-vigorous levels of RT (MVRT) had significantly greater positive effects on muscle mass, physical function, and lower limb strength compared to light-to-moderate (LMRT) exercise levels [12]. Chen classified MVRT as scores of 14-17 on the Borg Rating of Perceived Exertion (RPE) scale, 7-8 on the modified Borg’s scale, or 70-84% of one-repetition maximum (1RM) [12]. The Borg RPE reliably reflects heart rate, while the modified Borg’s scale reflects perceived breathlessness [15, 16]. The 1RM, the maximum weight lifted once, reliably measures muscle strength regardless of gender or muscle group [17]. The LMRT level is characterized by scores of 6-11 on the Borg RPE scale, 0-4 on the modified Borg’s scale, or less than 49% of 1RM. Most MVRT programs focus on gait and balance with strengthening of the lower limbs due to the recognized benefits in reducing falls, overall mortality, and decline in instrumental activities of daily living [12]. Both moderate and MVRT levels were associated with improvements in static and dynamic balance and overall functional mobility [12]. This is manifested in a significant reduction in time to complete the Timed Up and Go (TUG) test and more repetitions in the 30-second chair stand test (p < 0.05). However, no effect was observed in the five-times sit-to-stand (5TSTS), Short Physical Performance Battery (SPPB), and gait speed assessments. There is a significant increase in leg press strength, but no increase in skeletal muscle mass or handgrip strength (p < 0.05). However, the heterogeneity of interventional exercise protocols and nutritional support identified in this meta-analysis complicates clinical application. Different randomized controlled trials (RCTs) have supported the benefits of structured RT programs in different dimensions. Ramírez-Campillo’s study demonstrated that a 12-week high-speed RT program (70-minute sessions, three times per week) optimized muscle power and functional task performance in elderly women [18]. The training exercises included bench press, standing upper row, leg press, and extension. The slow-speed training cohort performed three sets of eight repetitions for each exercise at 75% of their baseline 1RM, with a one-minute inter-set rest period. The high-speed group completed all repetitions using a concentric muscle action as fast as possible and took three seconds for the eccentric muscle action. Additionally, the high-speed group completed two sets of five repetitions of medicine ball throwing (2 kg) and two sets of three repetitions of countermovement jumps in each session. Overall, there was a significant improvement in ball throwing performance, 10-m walking sprint, and 8-foot up-and-go test in the high-speed group compared to the low-speed RT protocol. Another RCT revealed significant gains in muscle mass and functional strength in 70-year-old individuals following a 10-week progressive high-intensity program, facilitated by two instructors and consisting of three 45-minute sessions per week [8]. This involved a progressive increase in the number of sets, up to four sets of 10-12 repetitions, emphasizing concentric and eccentric muscle contractions lasting approximately two seconds each, with faster contractions. To maintain a minimum self-reported modified Borg’s scale of 6 among participants, more weight was progressively added. Resistance bands, weighted vests, belts, and backpacks (filled with weights or water bottles) were provided. An oral nutritional supplement (ONS) was offered once daily but was not a mandatory component of the program. Overall, there was a significant improvement in the total SPPB score [19], TUG time, and handgrip strength compared to the control group, which was asked to go about their normal lives.

For individuals unable to perform high-load exercises, low-load RT combined with blood flow restriction (BFR) (20-50% 1RM) has emerged as an effective alternative, promoting muscle hypertrophy and strength gains with minimal mechanical load in the elderly [20]. Both Lixandrão ME et al. and Centner C et al., in their respective systematic reviews and meta-analyses, demonstrated that BFR training yields similar improvements in muscle mass compared to high-load RT, although it appears less effective for enhancing muscle strength regardless of occlusion pressure and cuff width [21, 22]. Both papers included studies spanning a four- to twelve-week timeframe, focusing predominantly on leg press, knee extension, and chest press exercise regimens.

Since the COVID-19 pandemic, home-based prehabilitation programs have gained popularity due to improved accessibility and higher reported adherence [23]. However, skepticism and criticism of the home-based approach stem largely from a lack of clarity concerning its safety and efficacy [24]. Furthermore, the reported rates of non-adherence and patient dropout in facility-based prehabilitation programs may be underestimated [18]. Older adults with sarcopenia often exhibit impaired balance and mobility, increasing their risk of injury during exercise. This underscores the need for multidisciplinary involvement, including physiotherapists and exercise physiologists, to ensure appropriate supervision, exercise modification, and injury prevention strategies.

Another key logistical barrier is the limited surgical scheduling window, typically only three to four weeks for elective procedures, whereas most exercise interventions require ten to twelve weeks to achieve clinically significant benefits [12]. The potential role of shorter-duration RT programs remains under investigation. It remains unknown if short-duration RT programs can allow older people to become familiar with the habit of exercising and enable them to progress in their resistance. In one RCT, three to five days of tailored exercise therapy during hospitalization improved functional outcomes (SPPB score and gait velocity), but did not increase muscle mass [25].

Nutrition Optimisation

There is evidence suggesting that the older population with sarcopenia tends to consume less dietary protein daily, mainly due to reduced energy needs secondary to diminished muscle regeneration and accelerated muscle protein breakdown [26, 27]. This remains a serious concern, as only approximately 14-30% of older people consume an adequate amount of protein [28, 29]. A summary of nutrients shown to be beneficial in sarcopenia management, as compiled by the European Society for Clinical and Economic Aspects of Osteoporosis, Osteoarthritis and Musculoskeletal Diseases (ESCEO working group), includes whey protein, leucine, methyl β-hydroxy β-butyrate, vitamin D, and various antioxidants [26]. Studies indicate whey protein's exceptional efficacy in stimulating systemic muscle protein anabolism, attributed to its rapid absorption, high digestibility, and rich amino acid content [29, 30]. Multiple studies have illustrated that it promotes muscle health in sarcopenic elderly individuals [30, 31]. A meta-analysis by Huang LP et al. indicated inferior effects of whey protein supplementation on RT-induced gains in lean body mass compared to milk protein supplementation in adults aged 60 and above [32]. A daily intake of two cups of milk, in conjunction with RT, was found to be an effective strategy against muscle loss, resulting in a mean increase of 0.31 kg in lean muscle mass (p < 0.05) [32].

In the surgical context, addressing pre-operative protein requirements is critical to counteract the catabolic effects of illness and to support tissue repair and recovery [33]. A systematic review conducted by Gillis C et al. demonstrated that unimodal nutrition prehabilitation significantly shortened the length of stay by two days, independent of exercise therapy, after colorectal surgery [34]. The included nine studies offered a minimum seven-day pre-operative dietary regimen, involving either 400 ml of Fortisip daily or achieving a minimum daily protein intake of 1.2 g/kg of ideal body weight, with or without optional nutritional counselling. These studies reported pre-operative nutritional intake ranging from 88 to 425 kcal per day and protein intake between 18 and 22 grams, over durations spanning 14.5 to 37.6 days [34]. Another meta-analysis conducted by Yoshimura Y et al. revealed limited but notable improvements in sarcopenia and physical frailty management through protein supplementation, specifically enhancing muscle mass and handgrip strength [26]. The optimal protein dosage, however, is not definitively established; a review of thirteen RCTs showed substantial heterogeneity in intervention dosages, ranging from 10 to 30 grams per day, in the form of whey protein, milk supplements, and ricotta cheese [27]. However, another meta-analysis focusing on patients with colorectal cancer undergoing surgery did not demonstrate a significant reduction in the overall complication rate with pre-operative nutrition therapy [35]. Each study supplied a liquid oral supplement, primarily carbohydrate-based (approximately 50%), with daily dosages ranging from 400 to 1000 ml. Program lengths varied across studies: three provided supplements five days before surgery, while three others extended from cancer diagnosis to post-surgery. Overall, data inconsistencies make recommendations contentious.

Vitamin D deficiency is associated with poorer lower extremity function among older patients [26]. Muir's meta-analysis of thirteen vitamin D supplementation trials (excluding exercise interventions) in older adults demonstrated that daily doses of 800-1000 IU yielded improvements in muscle function, as evidenced by reduced postural sway, faster TUG test completion times, and increased lower extremity strength [35]. A meta-analysis conducted by Beaudart C et al., involving 29 vitamin D supplementation trials, revealed a marginal positive correlation between supplementation and muscle strength among vitamin D-deficient older adults (p = 0.05); however, a dose-response relationship was not established due to variations in study protocols [35].

A modern dietary protocol for sarcopenic patients undergoing prehabilitation should be tailored but guided by evidence-based macronutrient and micronutrient targets. Currently, the European Society for Clinical Nutrition and Metabolism (ESPEN) recommends a protein intake of 1.0-1.2 g/kg body weight/day for older adults [29]. Combining increased protein consumption with regular exercise is the most successful method for optimising muscle growth [3, 33]. Therefore, prehabilitation programs integrating nutritional supplements with exercise might demonstrate a synergistic effect that translates into better recovery. While food should be the primary delivery mode, ONS and specialised formulas (e.g., whey protein, vitamin D supplementation) are frequently needed to meet intake goals, especially in patients with reduced appetite or high metabolic demand [26]. The high prevalence of malnutrition currently observed among the sarcopenic population highlights the urgent need to ensure all older adults are supported effectively to achieve sufficient nutritional intake. Effective implementation requires multidisciplinary collaboration to align nutrition with surgical timelines: dietary assessments and meal plans must be individualised by a registered dietitian; a physiotherapist should align exercise programs with dietary goals; and a pharmacist should evaluate potential nutrient-drug interactions, especially in patients taking multiple medications.

Psychological Support

Contemporary prehabilitation programs, in addition to physiological optimisation, have also incorporated psychological support for patients. This is particularly relevant in the field of surgical oncology, where it is estimated that concurrent psychological distress may affect over half of the patients [36]. Psychological support has also been shown to significantly enhance patients' motivation to follow exercise and nutritional recommendations [37]. Therefore, prehabilitation programs should include a screening process to identify key psychological comorbidities, including anxiety and depression, through validated evidence-based tools such as the Generalized Anxiety Disorder-7 (GAD-7), Kessler-10 (K-10), and the Patient Health Questionnaire-9 (PHQ-9). Following a positive screen, key interventions that have demonstrated evidence in improving psychological outcomes include stress management workshops, information sessions leading up to surgery, and brief counselling sessions to provide emotional support [36]. Despite this, there is currently no standardised method for psychological intervention, with variable frequency of sessions, intervention designs, and outcomes reported in the literature [36].

Management of Chronic Inflammation

The link between chronic inflammation and sarcopenia is well-established and is believed to be due to elevated levels of pro-inflammatory cytokines such as tumour necrosis factor-alpha (TNF-alpha), and interleukins 1 and 6 (IL-1 and IL-6) [38]. Underlying chronic inflammation and age-related increases in pro-inflammatory mediators exacerbate muscle wasting and weakness by inhibiting protein synthesis and promoting degradation, exerting a particularly significant effect on elderly patients at high risk of sarcopenia [38, 39]. This condition of chronic, low-level inflammation is hypothesised to originate from an increased number of cells exiting the cell cycle and entering senescence, subsequently developing a senescence-associated secretory phenotype and thereby inducing the production of pro-inflammatory cytokines, including TNF-alpha, IL-1, and IL-6 [38].

Glucocorticoids have been explored as a possible pharmacological agent to manage chronic inflammation [38]. While they function as potent immunosuppressors, thereby preventing the negative effects of cytokines, prolonged exposure to glucocorticoids may further contribute to sarcopenia through separate pathways that inhibit protein synthesis and promote protein breakdown. Some studies have explored the potential of using glucocorticoids in a controlled manner to enhance surgical outcomes in prehabilitation. The concept of augmented prehabilitation includes the potential use of glucocorticoids and anabolic steroids to improve surgical outcomes by mitigating acute inflammatory responses without causing long-term muscle damage [40]. This approach suggests that, when used judiciously, glucocorticoids might help manage inflammation during critical periods around surgery, potentially improving recovery and functional outcomes [40].

In addition to glucocorticoid therapy, there should be a multi-faceted approach to managing chronic inflammation in sarcopenic patients. Nutritional therapies such as whey protein supplementation and vitamin D repletion, as outlined earlier, play a role in modulating pro-inflammatory responses associated with sarcopenia [38, 41]. Current evidence suggests that while glucocorticoids may be beneficial in acute settings, their chronic use should be carefully monitored to avoid exacerbating sarcopenia. A more promising solution appears to be a balanced approach that combines pharmacological interventions with straightforward nutritional strategies and resistance exercises, as previously discussed.

Management of Hormonal Balance

Physiological mechanisms required to maintain appropriate muscle protein turnover (synthesis and degradation) involve a complex interplay between hormones such as growth hormone (GH), testosterone, cortisol, and insulin. Dysregulation of these pathways plays a critical role in the development of sarcopenia [42].

Studies have shown that reduced GH and insulin-like growth factor 1 levels are associated with decreased muscle mass and increased body fat, contributing to sarcopenia [42]. Efforts to treat muscle loss through GH injections have yielded mixed results, with some studies reporting increased muscle mass but not necessarily enhanced muscle strength [38]. Another important hormone to consider is cortisol, which can be elevated due to chronic stress, certain medical conditions, and ageing. Cortisol exerts catabolic effects through its action on the hypothalamic-pituitary-adrenal axis. Implementation of resistance exercises may help achieve hormonal balance, presenting a holistic strategy to counteract hypercortisolism and sarcopenia [43].

Insulin resistance presents another challenge in managing age-related hormonal dysfunction [38]. This hormone not only plays a vital role in glucose metabolism but also has an anabolic effect on muscles by promoting protein synthesis. When this pathway is impaired by muscular insulin resistance, it leads to decreased muscle mass and significantly contributes to sarcopenia [44]. This highlights yet another area where establishing a regular exercise routine, combined with targeted nutritional supplementation, can have a meaningful impact in reducing the risk of sarcopenia [45].

Challenges to implementation

Several challenges must be addressed to optimise rehabilitation programs for adults with sarcopenia. Currently, there are no nationally endorsed protocols specifically for identifying and managing sarcopenia preoperatively on an international scale. Sarcopenia is often underdiagnosed or diagnosed late, limiting the time available for meaningful prehabilitation. Although the European Society for Clinical Nutrition and Metabolism (ESPEN) and the EWGSOP, along with other surgical organisations, advocate for sarcopenia screening, its implementation remains inconsistent across public and private healthcare facilities and various surgical specialties. Additionally, there is no consensus on the most effective exercise program for pre-operative patients.

## Conclusions

Sarcopenia presents a significant and often under-recognised risk factor for adverse surgical outcomes, particularly in older adults and individuals with cancer or chronic illness. Despite its well-established impact on peri-operative morbidity and mortality, there remains a lack of standardisation in how sarcopenia is identified and managed prior to surgery. This review has outlined current evidence supporting further research into a standardised, multimodal approach to prehabilitation, encompassing resistance training, targeted nutritional strategies, psychological support, inflammation control, and hormonal regulation. The heterogeneity of current practices underscores the need for integrated, multidisciplinary prehabilitation programs that are both evidence-based and adaptable to individual patient needs and surgical timelines.

To improve outcomes and reduce peri-operative risk in this vulnerable population, future efforts must focus on creating standardised protocols, promoting early screening, and advocating for broader access to prehabilitation services, especially in underserved or resource-limited settings. As surgical demand grows alongside an ageing population, optimising care for sarcopenic patients is not only clinically necessary but also a vital component of sustainable, high-quality peri-operative care.
